# Low-Grade Systemic Inflammation Interferes with Anabolic and Catabolic Characteristics of the Aged Human Skeletal Muscle

**DOI:** 10.1155/2021/8376915

**Published:** 2021-12-07

**Authors:** Dimitrios Draganidis, Athanasios Z. Jamurtas, Niki Chondrogianni, George Mastorakos, Tobias Jung, Tilman Grune, Constantinos Papadopoulos, Konstantinos Papanikolaou, Ioannis Papassotiriou, Nikoletta Papaevgeniou, Athanasios Poulios, Alexios Batrakoulis, Chariklia K. Deli, Kalliopi Georgakouli, Athanasios Chatzinikolaou, Leonidas G. Karagounis, Ioannis G. Fatouros

**Affiliations:** ^1^Department of Physical Education and Sport Science, University of Thessaly, Karies, Trikala 42100, Greece; ^2^Institute of Chemical Biology, National Hellenic Research Foundation, 116 35 Athens, Greece; ^3^Unit of Endocrinology, Diabetes Mellitus and Metabolism, Aretaieion Hospital, School of Medicine, National and Kapodistrian University of Athens, Athens, Greece; ^4^Department of Molecular Toxicology, German Institute of Human Nutrition Potsdam-Rehbruecke (DIfE), 14558 Nuthetal, Germany; ^5^German Center for Diabetes Research (DZD), 85764, Muenchen-Neuherberg, Germany; ^6^German Center for Cardiovascular Research (DZHK), 10117 Berlin, Germany; ^7^First Department of Neurology, University of Athens, School of Medicine, Aeginition Hospital, Athens, Greece; ^8^Department of Clinical Biochemistry, “Aghia Sophia” Children Hospital, Athens, Greece; ^9^Department of Nutrition and Dietetics, University of Thessaly, Karies, Trikala 42100, Greece; ^10^Democritus University of Thrace, School of Physical Education and Sport Sciences, Komotini 69100, Greece; ^11^Nestlé Health Science, Translational Research, Vevey, Switzerland; ^12^Institute of Social and Preventive Medicine, University of Bern, Bern, Switzerland

## Abstract

Aging is associated with the development of chronic low-grade systemic inflammation (LGSI) characterized by increased circulating levels of proinflammatory cytokines and acute phase proteins such as C-reactive protein (CRP). Collective evidence suggests that elevated levels of inflammatory mediators such as CRP, interleukin-6 (IL-6), and tumor necrosis factor *α* (TNF-*α*) are correlated with deteriorated skeletal muscle mass and function, though the molecular footprint of this observation in the aged human skeletal muscle remains obscure. Based on animal models showing impaired protein synthesis and enhanced degradation in response to LGSI, we compared here the response of proteolysis- and protein synthesis-related signaling proteins as well as the satellite cell and amino acid transporter protein content between healthy older adults with increased versus physiological blood hs-CRP levels in the fasted (basal) state and after an anabolic stimulus comprised of acute resistance exercise (RE) and protein feeding. Our main findings indicate that older adults with increased hs-CRP levels demonstrate (i) increased proteasome activity, accompanied by increased protein carbonylation and IKK*α*/*β* phosphorylation; (ii) reduced Pax7^+^ satellite cells; (iii) increased insulin resistance, at the basal state; and (iv) impaired S6 ribosomal protein phosphorylation accompanied by hyperinsulinemia following an acute RE bout combined with protein ingestion. Collectively, these data provide support to the concept that age-related chronic LGSI may upregulate proteasome activity via induction of the NF-*κ*B signaling and protein oxidation and impair the insulin-dependent anabolic potential of human skeletal muscle.

## 1. Introduction

Chronic low-grade systemic inflammation (LGSI) develops with increasing age and is characterized by increased levels of circulating proinflammatory cytokines and acute phase proteins [1]. This age-associated LGSI represents an aging contributor and a leading cause of several age-related diseases and geriatric syndromes [2]. Moreover, LGSI has been conceptualized to interfere with muscle health by representing the mechanistic link between aging, skeletal muscle loss, and sarcopenia [3]. Indeed, compelling evidence indicates that increased levels of circulating C-reactive protein (CRP) and proinflammatory cytokines are associated with lower muscle mass and strength and increased risk of sarcopenia in older adults [3, 4].

Aged rats with elevated plasma levels of proinflammatory mediators demonstrated a blunted postprandial muscle protein synthesis (MPS) [5] whereas attenuation of LGSI using an anti-inflammatory treatment restores MPS to food intake [6]. Furthermore, the transcription factor nuclear factor-kappa B (NF-*κ*B) plays a pivotal role in muscle atrophy, as its activation induces profound muscle loss in mice by upregulating the ubiquitin proteasome system- (UPS-) mediated proteolysis [7, 8]. This scenario has been verified by studies using myotudes, revealing that proinflammatory cytokines are capable of inducing protein degradation and consequently skeletal muscle loss via a NF-*κ*B–dependent activation of the UPS [7–9].

However, the relative importance of chronic LGSI in protein synthesis and degradation is still poorly investigated in the aged human skeletal muscle and considerable gaps exist on the mechanistic link between LGSI and muscle anabolic and catabolic phenotype. The only two human studies that attempted recently to examine whether LGSI impairs the anabolic signaling and reduces postprandial MPS in older adults did not show any effect [10, 11]. Conversely, a previous report has shown that elevated plasma CRP and interleukin-6 (IL-6) levels were related to blunted postabsorptive muscle fractional synthesis rate in both young and older individuals [12].

In the present investigation, we provide a human clinical trial comparing the function of proteasome, the satellite cell and amino acid transporter protein content, and phosphorylation and protein content of signaling proteins related to protein synthesis and ribosome biogenesis in skeletal muscle, between healthy older men with elevated hs-CRP (indicative of elevated LGSI) and their age-matched counterparts with physiological hs-CRP levels. Comparison was performed at basal state and 3 hours after an acute anabolic stimulus consisting of a resistance exercise (RE) bout combined with protein feeding.

## 2. Methods

### 2.1. Participants

Forty-four healthy older men aged 65-75 years were initially recruited and underwent medical screening. Those who were nonsmokers, free of chronic disease (i.e., cancer, metabolic syndrome, cardiovascular, neurological, pulmonary, or kidney disease), inflammatory disease (i.e., osteoarthritis or rheumatoid arthritis), diabetes mellitus, and uncontrolled hypertension and had not used antibiotics or other medication that could interfere with the inflammatory status (i.e., corticosteroids) prior to the study underwent a baseline assessment after providing a written informed consent.

**Figure 1:**
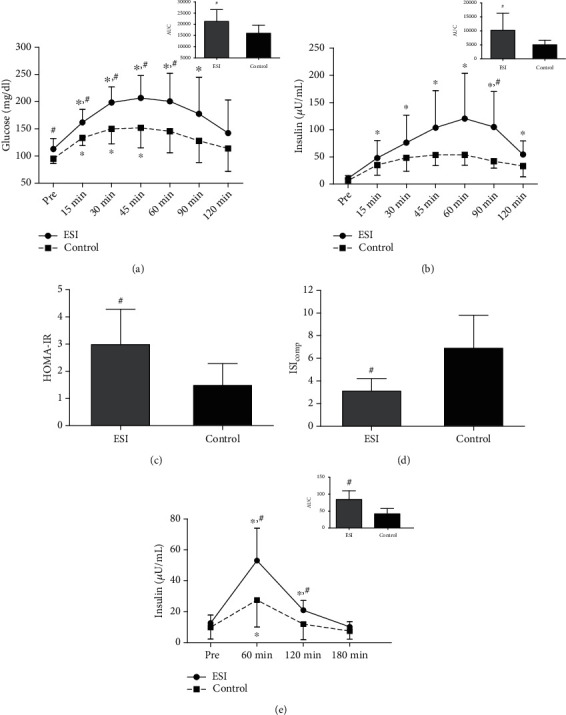
Chronic LGSI is associated with insulin resistance in healthy older adults. (a) Plasma glucose levels and quantification of the area under the curve (AUC) during the OGTT (*N* = 10 in ESI and 7 in control). (b) Plasma insulin levels and quantification of the AUC during the OGTT (*N* = 10 in ESI and 7 in control). Calculation of HOMA-IR (c) and ISIcomp (d) during the OGTT (*N* = 10 in ESI and 7 in control). (e) Plasma insulin levels and quantification of the AUC during the experimental day (*N* = 8 in ESI and 9 in control). ^∗^Significant difference from Pre (*p* < 0.05). ^#^Significant difference between groups (*p* < 0.05). All data are presented as mean ± SD.

Baseline assessment included blood sampling, to assess levels of systemic inflammation, measurement of body height, body mass, body composition, maximum strength, and physical performance (via the short physical performance battery (SPPB)) and determination of sarcopenia status, according to procedures described previously [13]. Furthermore, their daily habitual PA and macronutrient intake over a 7-day period were assessed via accelerometry and food diaries, respectively, as described [13]. Because both sarcopenia [14] and PA [15] levels may affect the molecular pathways linking LGSI and protein metabolism in skeletal muscle (the primary outcome investigated in this study), participants characterized as sarcopenic and/or had low or extremely high levels of PA were excluded from the study. Our aim was to include two groups of nonsarcopenic participants, with as much as possible similar PA level that would only differ in the inflammatory status. Accordingly, 10 older adults with plasma hs‐CRP > 1 mg/L (defined as elevated systemic inflammation; ESI), but otherwise healthy, were included in this clinical trial and compared with 11 healthy, age-matched individuals with hs-CRP < 1 mg/L (defined as physiological inflammatory status and served as the control; control) (S-Figure 1). All procedures were in accordance with the 1975 Declaration of Helsinki (as revised in 2013) and approved by the Institutional Review Board of the University of Thessaly. The study was preregistered at http://ClinicalTrial.gov/ (ID: NCT03308747).

### 2.2. Study Design

**Figure 2:**
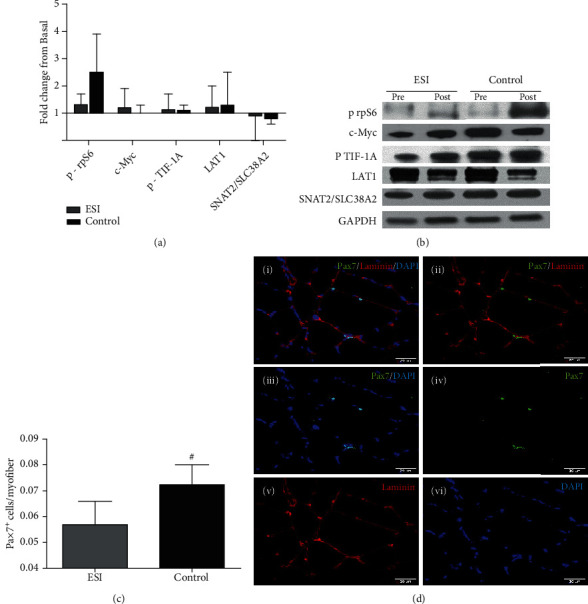
Evidence for impaired anabolic potential in older adults with chronic LGSI. (a) Quantification of phosphorylated ribosomal protein S6 (rpS6) (*N* = 8 in ESI and 10 in control), c-Myc (*N* = 5 in ESI and 6 in control), phosphorylated transcription initiation factor-1A (TIF-1A) (*N* = 7 in ESI and 7 in control), L-type amino acid transporter 1 (LAT1) (*N* = 7 in ESI and 9 in control), and sodium-coupled neutral amino acid transporter 2 (SNAT2/SLC38A2) (*N* = 7 in ESI and 9 in control) by densitometry (all results are expressed as ratio of each protein to GAPDH). (b) Representative western blots for rpS6, c-Myc, TIF-1A, LAT1, and SNAT2/SLC38A2. (c) Quantification of the Pax7^+^ cells per myofiber in skeletal muscle (*N* = 6 in ESI and 8 in control). (d) Representative images of staining against Pax7 antibody (i: Pax7/Laminin/DAPI stain of a muscle cross-section; ii–vi: channel views of merge Pax7/Laminin (ii), Pax7/DAPI and single Pax7 (iv), Laminin (v), and DAPI (vi). ^#^Significant difference between groups (*p* < 0.05). All data are presented as mean ± SD.

Following baseline assessment and one week prior to the experimental day, participants undertook an oral glucose tolerance test (OGTT). Then, they were asked to abstain from any type of strenuous PA over the 1-week period preceding the experimental day. During the experimental day, a resting blood sample and a muscle biopsy were taken after an overnight fast (basal state), and subsequently, participants performed an acute RE protocol and consumed a protein supplement as a single bolus immediately post-exercise. Blood samples were obtained at 30 min intervals during the 3-hour postprandial period while a second muscle biopsy was sampled at 3 hours after protein ingestion (S-Figure 2).

### 2.3. Oral Glucose Tolerance Test (OGTT)

A 2-hour 75 g OGTT was performed after an overnight fast with venous blood samples (~5 mL) received immediately before glucose loading (0 min) and at 15, 30, 45, 60, 90, and 120 min postloading to measure plasma glucose and insulin [16]. The homeostasis model assessment of insulin resistance (HOMA-IR) and the composite insulin sensitivity index (ISI_comp_) were calculated as described [16, 17].

### 2.4. Exercise and Protein Supplementation Protocols

Participants performed an acute RE bout consisted of eight sets of 10 repetitions/set of bilateral leg extension exercise on a leg extension machine (Cybex-VR2, Cybex International Inc., USA), at 70% of their maximal strength (1-repetition maximal, 1-RM) using a 2 min of rest between each set. Participants' maximal strength was determined at baseline using the 1-RM testing procedure on the leg extension machine, as described [18]. Prior to 1-RM testing, all participants were familiarized with the leg extension machine and were instructed the proper exercise execution.

A protein beverage that contained 0.4 g of whey protein isolate (Instantized BiPRO® I.P., Davisco Foods International, INC, Minnesota, USA) per kg of body mass was dissolved in 200 mL of water (without additives) and ingested as a single bolus immediately postexercise, to achieve maximal stimulation of the postprandial anabolic response [19]. The whey protein isolate powder consisted of 380 kcals, 91 g of whey protein, 1.3 g of fat, <0.2 g of lactose, and 8.5 g of other components (moisture and ash) per 100 g. It also provided 12.7 g leucine, 5.6 g isoleucine, and 5.4 g valine (49.3 g of essential amino acids per 100 g).

### 2.5. Blood Sampling and Assays

Baseline resting blood samples for the measurement of hs-CRP were collected twice, on separate days (1 week apart) and at the same time of day (between 07:00 and 09:00 am) to prevent circadian rhythm variations. All blood samples (baseline and those received during the OGTT and on the experimental day) were drawn from an antecubital arm vein while subjects were seated. For serum separation, blood samples were initially allowed to clot at room temperature and subsequently centrifuged (15,000xg, 15 min, 4°C). For plasma separation, a blood portion was collected into EDTA tubes and centrifuged immediately (1,370xg, 10 min, 4°C). Serum hs-CRP was quantitatively measured using a latex-enhanced immunoturbidimetric assay on a Roche Cobas 6000 clinical chemistry analyzer. The detectable limit was 0.01 mg/dL, and the interassay coefficient was one standard deviation (1 SD). Plasma glucose concentration was measured on a Clinical Chemistry Analyzer Z1145 (Zafiropoulos Diagnostica S.A., Koropi, Greece) using a commercially available kit (Zafiropoulos Diagnostica S.A.). Serum insulin concentrations were measured by means of electrochemiluminescence immunoassay using a Cobas e411 immunochemistry analyzer (Roche Diagnostics, Basel, CH) with intra- and interassay coefficients of variation (CV) <2.0%.

### 2.6. Muscle Biopsy Sampling

Muscle biopsy samples were obtained from the middle portion of vastus lateralis under sterile procedures and administration of local anesthetic (xylocaine 1%), using the Bergstrom needle (5-mm) technique with suction, as previously described [20]. Each biopsy provided 100-120 mg of muscle tissue of which a specimen of ~100 mg was rapidly frozen in liquid nitrogen and stored at -80°C for further analysis, whereas a portion of ~20 mg suited for histochemical analysis was aligned, placed in optimal cutting temperature (OCT) compound (Tissue-Tek), frozen in liquid nitrogen-cooled isopentane, and stored at -80°C until sectioned.

### 2.7. Proteasome Peptidase Assay and Immunoblot Analysis

Proteasome chymotrypsin-like (CT-L) and trypsin-like (T-L) activities were directly assayed with hydrolysis of the fluorogenic peptide LLVY-AMC (Enzo Life Sciences) and LSTR-AMC (Enzo Life Sciences), respectively, as previously described [20]. Briefly, muscle samples were homogenized in lysis buffer and the lysate was centrifuged (13,000 rpm, 10 min. 4°C). Total protein concentration was determined using the Bradford assay (Bradford Protein Assay, Bio-Rad). Proteasome CT-L and T-L activities were measured in 5-plicates of 20 *μ*g total protein after incubation (30 mi, 37°C) with diluted substrates LLVY-AMC and LSTR-AMC, respectively, in the presence of a specific proteasome inhibitor (MG132, 20 *μ*M). Fluorescence was measured using a VersaFluor™ (Bio-Rad) fluorescence spectrophotometer.

Protein expression levels of the proteasome (*β*5) and immunoprotesome (*β*1i, *β*2i, and *β*5i) subunits, phosphorylated ribosomal protein S6 (rpS6), amino acid transporters (SNAT2/SLC38A2 and LAT1), and ribosome biogenesis indicators (c-Myc and phosphorylated TIF-1A) were analyzed by immunoblotting [20]. Briefly, equal amounts (20 *μ*g) of protein were loaded into each lane, separated by SDS-PAGE electrophoresis (gradient precast gels were utilized (Mini-PROTEAN TGX Gels; Bio-Rad) and transferred to a nitrocellulose membrane (Trans-Blot® Turbo™ Transfer System; Bio-Rad) that was immediately blocked with 5% non-fat milk/Tris-buffered saline (1 h, room temperature). All primary antibodies were incubated overnight at 4°C while secondary antibodies were incubated for 1 h at room temperature (S-Table 1). Following incubation with antibodies, membranes were washed with Tris-bufferd saline-Tween (TBS-T) and enhanced chemiluminescence was utilized to detect the membrane-bound primary antibodies. Immunoblotting was also performed to detect carbonyl groups into proteins by utilizing the OxyBlot protein oxidation detection kit (EMD Merck Millipore–S7150).

### 2.8. Immunofluorescence

Muscle cross-sections (7 *μ*m) were prepared from OCT-embedded muscle samples and air-dried for 30 min before being stored at -80°C. For the 3-nitrotyrosine (3-NT), Nrf2 and phospho-IKK*α*/*β* localization, slides were fixed (4% paraformaldehyde, 10 min), blocked with a solution containing 2% BSA, 5% FBS, 0.2% Triton X-100, 0.1% sodium azide, and 5% GS in PBS, for 1 hour and incubated with appropriate primary and secondary antibodies (eTable 1) against specific antigens. Nuclei were stained with a fluorescent mounting medium containing DAPI prior to coverslipping. For the assessment of satellite cells (SC), slides were stained with appropriate primary and secondary antibodies (eTable 1) against specific antigens, as described [21]. Nuclei were visualized with a fluorescent mounting medium containing DAPI. For fiber typing and CSA analyses unfixed slides were incubated in a primary antibody cocktail (MHCII and laminin), followed by incubation with appropriate secondary antibodies. All slides were visualized using a confocal microscope (Zeiss LSM 700), and images were captured using the ZEN software with a 20x objective. 3-NT, Nrf2, and phospho-IKK*α*/*β* mean fluorescent intensity was assessed using ImageJ. Fiber typing, CSA, and SC analyses were conducted manually by the same blinded investigator. Special care was taken so that longitudinal fibers and areas of freeze-fractures were excluded from the analyses. Staining specificity was ensured using appropriate negative controls.

### 2.9. Statistical Analysis

Data normality was examined using the Shapiro-Wilk test (*n* < 21/group). Because most of our data was normally distributed, parametric statistics were utilized. Comparisons between ESI and control in all dependent variables at the basal state as well as in variables with a single measurement time point (i.e., glucose AUC, insulin AUC, HOMA-IR, ISI_comp_, or the fold change of all molecular parameters measured at 3 h following RE and protein ingestion) were examined using the independent samples *t*-test, whereas within group changes (basal vs. 3 h) were examined by using a paired-sample *t*-test. Different time point changes between ESI and control in glucose and insulin secretion during the OGTT and the experimental day were examined by using a two-way, repeated measure ANOVA with planned contrasts. A Bonferonni test was applied for post hoc analysis, when a significant interaction was detected. Correlations were performed using Pearson's correlation analysis. For all dependent variables, effect sizes (ES) and confidence intervals (CI) were calculated according to the corrected for bias Hedge's g method (eTable 2). ES was considered none, small, medium-sized, and large for values 0.00-0.19, 0.20-0.49, 0.50-0.79, and ≥0.8, respectively. Significance was set at *p* < 0.05. Data are presented as means ± SDs. The analysis was performed by utilizing the SPSS 20.0 software (IBM SPSS Statistics).

## 3. Results

### 3.1. Participants' Baseline Assessment

Participants' baseline characteristics are summarized in Table 1. Eleven control and 10 ESI participants were finally included in the analysis due to application of very strict inclusion criteria. The two groups differed significantly in the inflammatory status with hs-CRP levels being almost 3-fold higher in ESI compared to control (*p* = 0.009). The average hs-CRP in ESI was 2.3 mg/L that classifies them as being at an average risk for cardiovascular disease development [22]. Of the 11 individuals included in ESI, 10 had a hs-CRP value between 1 and 3 mg/L and only one individual had >3 mg/L (values that are consistently reported in studies examining the low-grade systemic inflammation in older adults [4, 10, 11, 23]). All participants were classified as nonsarcopenic, and the two groups were characterized by comparable BMI and body composition (fat mass, fat percent, fat free mass, and lean body mass). No differences were noted among groups in terms of physical performance (Table 1), habitual PA, and dietary profile (eTable 3). Altogether, the two groups had a similar anthropometric profile, PA, and dietary intake patterns with hs-CRP being the only variable actually discriminating them.

### 3.2. LGSI Is Associated with Insulin Resistance in Healthy Older Adults

**Figure 3:**
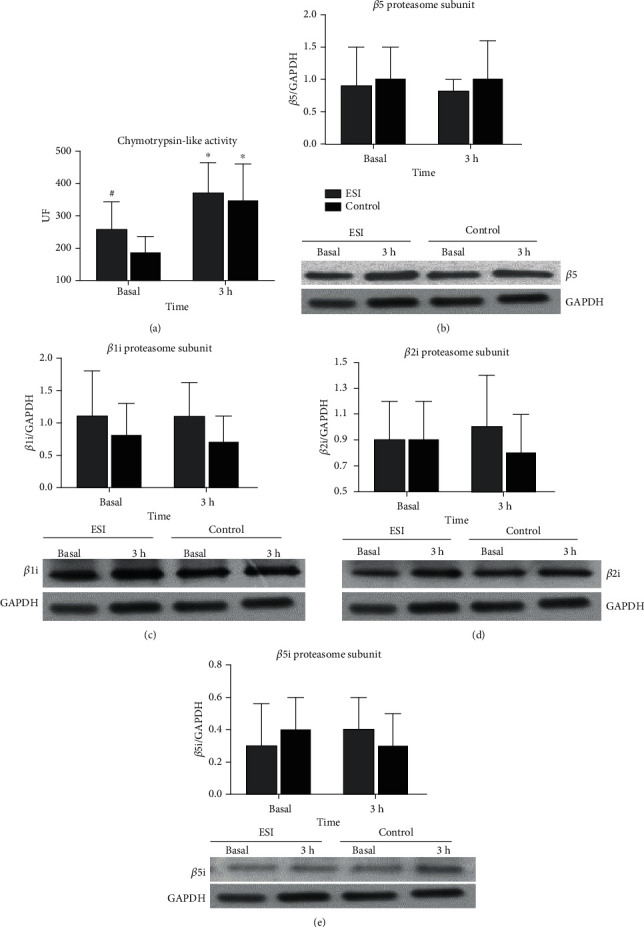
Increased proteasome activity at basal state in response to chronic LGSI in older adults. (a) Enzymtic determination of chymotrypsin-like proteasome activity (*N* = 9 in ESI and 10 in control). (b–e) Quantification of immunoproteasome (*β*1i, *β*2i, and *β*5i) and proteasome (*β*5) subunits by densitometry (results are expressed as ratio of each subunit to GAPDH) and representative western blots (*N* = 8 in ESI and 10 in control). ^∗^Significant difference from basal (*p* < 0.05). ^#^Significant difference between groups (*p* < 0.05). All data are presented as mean ± SD.

In light of the well-described association of insulin resistance with chronic LGSI [24] and its critical involvement in muscle protein metabolism [25, 26], we determined participants' response to an OGTT before the experimental day. As shown in Figures 1(a) and 1(b), ESI displayed increased fasting glycemia (+19%, *p* = 0.038) as well as increased glycemia and insulinemia throughout the OGTT. Both glucose and insulin area under the curve (AUC) were significantly greater in ESI compared to the control by 25% (*p* = 0.035) and 50% (*p* = 0.048), respectively (Figures 1(a) and 1(b)). Interestingly, glucose AUC was positively correlated with hs-CRP levels (*r* = 0.542, *p* = 0.025) (S-Figure 3 a). HOMA-IR was higher (*p* = 0.021) and ISI_comp_ lower (*p* = 0.002) in ESI (Figures 1(c) and 1(d)). Collectively, these findings clearly indicate that LGSI in ESI is associated with an increased insulin resistance compared to the control.

Moreover, on the experimental day, plasma insulin levels during the 3-hour postexercise period was significantly higher in ESI (*p* = 0.015-0.044) with the relevant AUC being 2-fold greater compared to the control (*p* = 0.001) (Figure 1(e)) whereas glucose levels remained unaltered in both groups. This finding is further supported by a positive correlation detected between hs-CRP levels and insulin AUC (*r* = 0.633, *p* = 0.005) (S-Figure 3 b).

### 3.3. Impaired S6 Ribosomal Protein Phosphorylation following RE and Protein Feeding under Conditions of LGSI

Considering the impaired postprandial MPS observed previously in aged rats with LGSI [5, 6], we investigated the ability of ESI and control to stimulate key molecules involved in anabolic signaling and ribosome biogenesis following an anabolic stimulus consisting of RE and protein ingestion. Phosphorylation of rpS6 (Ser^235/236^) increased by 1.5-fold in control and was greater than ESI (*p* = 0.030) (Figures 2(a) and 2(b)). In contrast, no changes were noticed in protein expression of c-Myc and phosphorylated TIF-1A (Ser^649^) in both groups, suggesting that ribosome biogenesis remained unaltered from basal state (Figures 2(a) and 2(b)). We next determined the relative protein content of muscle amino acid transporters LAT1 and SNAT2/SLC38A2, as a defective amino acid delivery in skeletal muscle has been reported to be a major cause of impaired anabolic signaling and consequently reduced MPS [27]. However, no time or group differences were observed in protein content of these amino acid transporters (Figures 2(a) and 2(b)).

### 3.4. LGSI Is Associated with Lower Muscle Content of Pax7^+^ Satellite Cells

**Figure 4:**
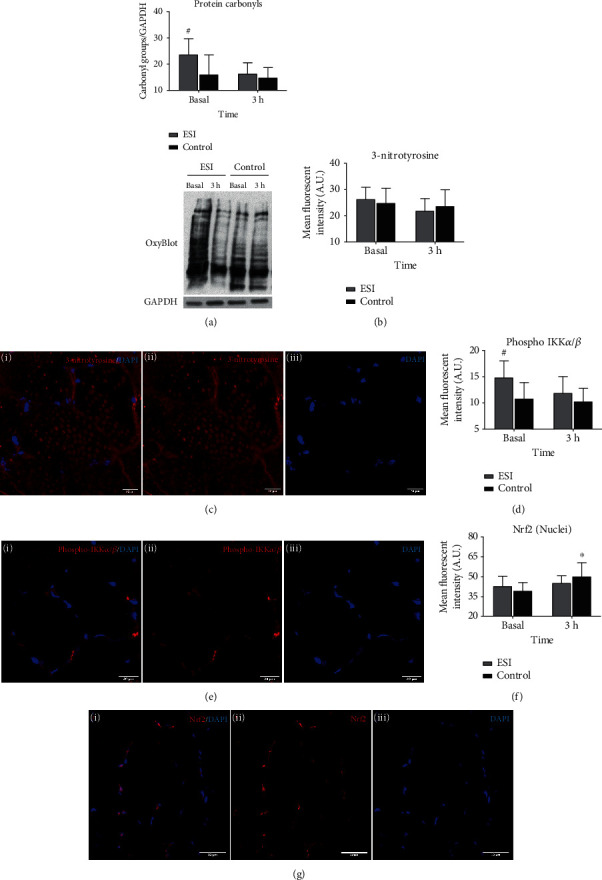
The increased proteasome activity at basal state coincides with increased protein carbonylation and IKK*α*/*β* phosphorylation. (a) Quantification of protein carbonyl groups (OxyBlot) by densitometry (results are expressed as ratio of protein carbonyl groups to GAPDH) and representative western blots (*N* = 9 in ESI and 11 in control). (b) Quantification of mean fluorescence intensity for 3-nytrotirosine and (c) representative images of 3-nitrotyrosine localization in skeletal muscle (i: 3-nitrotyrosine/DAPI stain of a muscle cross section; ii and iii: single channel views of 3-nitrotyrosine and DAPI) (*N* = 8 in ESI and 10 in control). (d) Quantification of mean fluorescence intensity for phosphorylated IKK*α*/*β* and (e) representative images of phosphorylated IKK*α*/*β* localization in skeletal muscle (i: phosphorylated IKK*α*/*β*/DAPI stain of a muscle cross section; ii and iii: single channel views of phosphorylated IKK*α*/*β* and DAPI) (*N* = 7 in ESI and 10 in control). (f) Quantification of mean fluorescence intensity for Nrf2 into the nulcei and (g) representative images of Nrf2 localization in skeletal muscle (i: Nrf2/DAPI stain of a muscle cross section; ii and iii: single channel views of Nrf2 and DAPI) (*N* = 8 in ESI and 9 in Control). ^∗^Significant difference from basal (*p* < 0.05). ^#^Significant difference between groups (*p* < 0.05). All data are presented as mean ± SD.

Previous studies in rodents reported that TNF-*α* promotes depletion of SC pool through apoptosis induction in the aged muscle [28] while treatment with an immunosuppressant drug enhances the number of Pax7^+^ cells and stimulates the formation of new myofibers [29]. Therefore, we hypothesized that chronic elevation in plasma hs-CRP levels might interfere with muscle SC content in older adults thus limiting muscle's anabolic potential. Consequently, muscle biopsies obtained at basal state were immunohistochemically stained and analyzed for Pax7^+^ cells and muscle fiber characteristics (S-Figure 4). Interestingly, more Pax7^+^ cells per myofiber were found in control compared to ESI (+21%, *p* = 0.004) (Figures 2(c) and 2(d)). The total number of fibers (both type I and type II), fiber distribution, total nuclei content, CSA (cross-sectional area) of type II fibers, and myonuclear domain of both type I and type II fibers were comparable among groups (eTable 4). A greater CSA in type I fibers was revealed in ESI compared to control (*p* = 0.019) (eTable 4), but it is the type II fibers that are more susceptible to atrophy with aging [30].

### 3.5. LGSI Interacts with Proteasome Activity in Skeletal Muscle

The UPS is one of the main proteolytic systems in skeletal muscle and experimental models revealed that it is predominantly involved in muscle atrophy induced by systemic inflammation [7–9]. Therefore, we compared proteasome activity and the expression of its catalytic constitutive *β* subunits and immunosubunits (*β*i) between ESI and control. ESI displayed higher basal CT-L proteasome activity than control by 40% (*p* = 0.022) (Figure 3(a)). A Pearson correlation analysis revealed that CT-L activity was positively correlated with plasma levels of hs-CRP (*R* = 0.576, *p* = 0.010) (S-Figure 5 a), suggesting that basal proteasome activity may be affected under conditions of LGSI.

At 3 h following RE and protein supplementation, CT-L activity increased in ESI and control by 59% (*p* = 0.037) and 95% (*p* = 0.001), respectively (Figure 3(a)). Although the induction in CT-L activity was greater in control by 35%, no statistically meaningful differences were detected between groups (*p* > 0.05). By performing a longitudinal analysis of both groups, we observed that the increase in CT-L activity following RE and protein feeding was inversely correlated with its basal levels (*R* = −0.616, *p* = 0.006) (S-Figure 5 b), which suggests a potential negative feedback loop between basal proteasome activity and its postexercise activation, i.e., the higher the activity prior to exercise the smaller its increase postexercise, an observation that should be further investigated. No time- or group-related differences were observed in T-L proteasome activity (S-Figure 5 c) and protein expression levels of the catalytic immunoproteasome subunits *β*1i, *β*2i, and *β*5i and the proteasome *β*5 subunit (Figures 3(b)–3(e)), suggesting that the observed differences in CT-L activity are solely related to alterations in proteasome activity and not to its protein expression.

### 3.6. Increased Protein Carbonyl Formation Coincides with LGSI

Given the observed differences in CT-L proteasome activity between ESI and Control, we then determined the levels of 3-NT and protein carbonyl via immunofluorescence and immunoblotting (Oxyblot), respectively, as indicators of muscle-derived oxidized proteins. The proteasome is activated in response to increased oxidative damage to proteins, to eliminate modified proteins and preserve protein homeostasis [31] thereby resulting in increased proteolysis in skeletal muscle [32].

The concentration of protein carbonyl groups at the basal state was higher in ESI compared to control by 47% (*p* = 0.026) (Figure 4(a)). However, there were no differences among groups at 3 h following RE and protein bolus ingestion, with ESI displaying a trend for reduced (by 26%, *p* = 0.058) protein carbonyl concentration compared to basal levels (Figure 4(a)) that might be attributed to its already elevated basal levels. No differences were detected in 3-NT levels among groups at any time point (Figures 4(b) and 4(c)), which is probably attributed to the fact that protein carbonyl formation in skeletal muscle is more abundant compared to other protein modifications induced by increased oxidation [31].

### 3.7. Evidence for Increased NF-*κ*B Activation at Basal State in Response to LGSI

Next, we examined the activation of the transcriptional factors NF-*κ*B and Nrf2 as both of them have been implicated in the regulation of the proteasome [7, 31]. We therefore assessed the levels of phosphorylated IKK*α*/*β*, as its phosphorylation dictates enhanced degradation of the I*κ*B complex thus leading to translocation of activated NF-*κ*B into the nucleus [7], while the Nrf2 activation was directly assessed through determination of its levels in the nucleus. A greater amount of basal phosphorylated IKK*α*/*β* was seen in ESI compared to control (+37%, *p* = 0.027) (Figures 4(d) and 4(e)), whereas no group differences were detected in terms of nuclear Nrf2 levels (Figures 4(f) and 4(g)). Three hours postexercise, both the phosphorylated levels of IKK*α*/*β* (Figures 4(d) and 4(e)) and nuclear Nrf2 levels (Figures 4(f) and 4(g)) were comparable among groups and unaltered from their basal levels.

## 4. Discussion

Previous *in vitro* and *in vivo* studies have identified that chronic LGSI inhibits MPS in the postprandial state [5, 6] and promotes proteasome-mediated proteolysis primarily through the activation of the transcriptional factor NF-*κ*B [7–9]. However, the mechanistic link between chronic LGSI and skeletal muscle loss remains elusive in the aged human skeletal muscle. Our aim was to compare the molecular background related to muscle protein metabolism among healthy older adults with elevated and physiological concentration of plasma hs-CRP. Therefore, proteasome activity and expression of its catalytic subunits, the content of Pax7^+^ SC, the amino acid transporter protein content, and the phosphorylation status and protein content of signaling proteins related to translation initiation and ribosome biogenesis were assessed in muscle biopsies collected from vastus lateralis before (preexercise, basal state) and 3 hours after an acute anabolic stimulus consisting of RE bout and a protein bolus ingestion. Our results support the idea that chronic LGSI promotes proteasome activity through activation of the NF-*κ*B signaling and reduces the insulin-dependent anabolic potential in the aged human skeletal muscle.

Basal proteasome activity was found profoundly elevated in ESI compared to the control, and it was positively correlated with plasma hs-CRP levels. This is consistent with the concentration-dependent protein degradation observed in differentiated myotubes following treatment with TNF-*α* [9], suggesting that the influence of chronic LGSI on proteasome activity is proportionate to the degree of inflammation. We also observed increased muscle protein carbonylation and a greater amount of phosphorylated IKK*α*/*β* in ESI compared to control, both of which may explain the enhanced proteasome activity in ESI. Indeed, previous *in vivo* and *in vitro* experiments revealed that muscle wasting under chronic inflammatory conditions results from increased proteasome activation that is predominantly regulated by the NF-*κ*B signaling pathway [8, 9]. For instance, treatment of cell lines with TNF-*α* resulted in NF-*κ*B activation and muscle protein loss [9] that was prevented when a specific NF-*κ*B inhibitor was applied [8]. Furthermore, RONS contribute to the accumulation of oxidized protein aggregates in cells by inducing oxidative modifications to proteins, the most abundant form of which are protein carbonyls [31]. Oxidized proteins are then targeted for proteolytic degradations through the UPS and lysosome system and, thus, are considered a principal activator of these major proteolytic systems [31]. Previous research highlighted that protein carbonylation increases with aging [33] and might contribute to skeletal muscle loss through stimulation of UPS-mediated protein degradation [34]. Although, the NF-*κ*B activation was indirectly assessed in this study, our findings provide insight of the crosstalk between chronic LGSI, protein carbonylation, NF-*κ*B signaling, and proteasome activity in the aged human skeletal muscle.

At 3 hours following the RE bout and protein bolus ingestion, proteasome activity was similarly elevated in the ESI and control groups, though no significant alterations in protein expression of its catalytic subunits, protein carbonyls levels, or phosphorylated levels of IKK*α*/*β* were noted. A few recent studies have reported that protein content and mRNA expression of markers related to UPS are similarly elevated in young and older individuals in response to acute RE [35] and that amino acid ingestion has no impact on these acute responses [36]. Here, we provide novel evidence that the increase in proteasome activity following RE combined with protein ingestion remains unaffected by chronic inflammatory status in healthy older adults.

An intriguing finding of this investigation is that phosphorylation of rpS6 was substantially attenuated in ESI compared to control at 3 hours following RE and protein feeding. Although, the role of rpS6 phosphorylation remains equivocal in terms of translational control [37], there is evidence showing that it is critically involved in regulating cell size [37]. In addition, rpS6 is one of the main substrates of ribosomal protein S6 kinase (S6K1), a downstream effector of mTOR, and its phosphorylation is inhibited following treatment with rapamycin (i.e. a potent mTORC1 inhibitor) [38], thus suggesting that rpS6 phosphorylation status might be used as an indirect measure of the mTOR-S6K1 signaling axis activation. Given that the latter is fundamental for contraction-induced stimulation of MPS following RE [26, 38] and that LGSI-driven insulin resistance is attributed to impaired activation of the IRS1-PI3K-PDK1-Akt signaling pathway [26, 39], we assume that the blunted rpS6 phosphorylation in ESI dictates reduced responsiveness of the PI3K/Akt/mTOR/S6K1 signal transduction pathway as a consequence of the coexistence of chronic LGSI and insulin resistance. Actually, ESI displayed insulin resistance during the OGTT and increased insulinemia during the 3-hour postprandial period following RE. Indeed, insulin resistance triggered by systemic inflammation has been implicated in the impaired activation of the PI3K/Akt/mTOR signaling pathway [26, 39]. Furthermore, our observation that increased protein carbonylation in the basal state was accompanied by impaired rpS6 phosphorylation during the postprandial period following RE in ESI is consistent with the concept that elevated RONS production, and consequently protein oxidation, not only affect proteasome-mediated proteolysis but also inhibit the activation of the Akt/mTOR signaling pathway, suggesting a redox-dependent regulation of the anabolic signaling in skeletal muscle [32]. Based on these findings, we propose that the coexistence of chronic LGSI-driven insulin resistance and protein oxidation contribute to the blunted stimulation of PI3K/Akt/mTOR/S6K1 signal transduction pathway, in the aged human skeletal muscle.

There were no differences in protein content of LAT1 and SNAT2/SLC38A2 between ESI and Control, either in the basal state or following RE and protein ingestion, suggesting that protein expression of amino acid transporters is not related to the inflammatory status. Similarly, we failed to detect any difference among groups in ribosome biogenesis, as the expression of c-Myc and phosphorylated TIF-1A was unaltered in response to RE and protein feeding. It has been shown that acute RE promotes phosphorylation of TIF-1A (Ser^649^) and c-Myc total protein expression, as early as 1-2 hours postexercise, in young males [40, 41]. However, the work by Stec et al. [42, 43] revealed that ribosome biogenesis is impaired in older adults following (24 hours) acute RE session as compared to the younger ones, but in response to chronic (4 weeks) RE training, there was a remarkable rise in rRNA production along with elevated c-Myc accumulation in older adults that presented a hypertrophic adaptation. Thus, whether ribosome biogenesis might occur during a longer recovery period (≥3 hours) and if LGSI has a potential role on this response warrants further investigation.

Beyond the metabolic processes of protein synthesis and degradation, SC are also considered a key determinant of skeletal muscle atrophy and hypertrophy through their regulatory role in myogenesis and muscle regeneration [30, 44]. *Τ*his study shows that aged individuals with elevated plasma hs-CRP levels are characterized by lower SC content compared to their control counterparts. At the same time, ESI displayed a greater CSA in type I fibers than control whereas CSA in type II fibers was comparable among groups. It appears, therefore, that the LGSI-associated reduction in SC pool during aging may be an early event preceding muscle atrophy. Previous work revealed that chronic inflammation driven by rheumatoid arthritis does not affect the SC content as well as their regenerative potential in skeletal muscle [45, 46]. However, rheumatoid arthritis is not indicative of LGSI seen in healthy aging, since patients usually demonstrate disparate inflammatory status (low to severe inflammation) and use medication that mitigates inflammation and consequently its potential impact on SC. A mechanism that has been described to negatively affect the abundance of SC and their function is that of the elevated levels of ROS in skeletal muscle along with a diminished antioxidant capacity, both associated with aging [44]. Accordingly, in this study, the increased hs-CRP was accompanied by elevated levels of protein carbonyls, a sign of increased protein oxidation in skeletal muscle.

However, the absence of direct measurement of skeletal muscle protein synthesis and breakdown rates represent a limitation in the present study which does not allow us to conclude on the effect of chronic LGSI on muscle protein turnover. The observed changes in signaling proteins and proteasome activity represent indirect markers of muscle protein turnover and, therefore, cannot be translated into altered rates of protein synthesis and breakdown.

## 5. Conclusions

In conclusion, our results highlight that healthy older adults with increased plasma hs-CRP levels display enhanced muscle proteasome activity in the basal state that relates to increased protein carbonylation and NF-*κ*B activation. They are also characterized by lower SC content and diminished capacity to stimulate the insulin-mediated activation of the PI3K/Akt/mTOR/S6K1 signal transduction pathway. Although the experimental design of the present study does not provide direct evidence on a casual relationship between chronic LGSI and altered muscle protein turnover (the study's primary objective was to compare muscle characteristics and protein turnover signaling among healthy older adults with disparate chronic LGSI levels), the findings build on the mechanistic rational that chronic LGSI might simultaneously affect cellular signaling related to skeletal muscle growth and atrophy [26] and provide directional evidence for future studies.

## Figures and Tables

**Table 1:** Participants' baseline characteristics.

Parameter	Control (*N* = 11)	ESI (*N* = 10)	*t-*test (*p* value)
Age (yrs)	67.9 ± 2.7	68.2 ± 3.4	0.917
Body composition			
Height (m)	1.72 ± 0.08	1.74 ± 0.05	0.480
Weight (kg)	81.6 ± 7.4	88.0 ± 4.7^∗^	0.029
BMI (kg/m^2^)	27.8 ± 2.3	29.3 ± 2.4	0.170
Fat mass (kg)	23.6 ± 5.2	26.6 ± 2.0	0.102
Fat (%)	30.2 ± 4.9	31.9 ± 2.4	0.331
Fat-free mass (kg)	56.9 ± 4.5	59.9 ± 4.6	0.134
Lean body mass (kg)	53.7 ± 4.3	56.7 ± 4.5	0.140
ALM (kg)	23.4 ± 2.1	25.3 ± 2.5	0.077
SMI (kg/m^2^)	8.0 ± 0.5	8.4 ± 0.9	0.160
Functional performance			
Grip strength (kg)	37.7 ± 3.5	37.5 ± 7.3	0.947
SPPB (score)	11.9 ± 0.3	11.5 ± 0.5	0.050
Inflammatory status			
hs-CRP (mg/L)	0.6 ± 0.2	2.3 ± 1.7^∗∗^	0.009
Physical activity			
Sedentary (min/day)	370.4 ± 82.9	361.7 ± 99.9	0.836
Light (min/day)	361.9 ± 104.3	368.7 ± 104.9	0.886
Moderate (min/day)	57.7 ± 22.3	45.3 ± 23.5	0.242
Vigorous (min/day)	5.9 ± 7.6	1.2 ± 3.1	0.092
MVPA (min/day)	63.6 ± 25.8	46.5 ± 25.1	0.150
Steps/day	9254.0 ± 2695.2	7367.9 ± 1729.2	0.079

Data are presented as mean ± SD. ALM: appendicular lean mass; SMI: skeletal muscle mass index; SPPB: short physical performance battery; hs-CRP: high sensitive CRP; MVPA: moderate-to-vigorous PA. ^∗^Difference between groups, *p* < 0.05; ^∗∗^difference between groups, *p* < 0.01.

## Data Availability

The data that support the findings of this study are available from the corresponding author upon reasonable request.

## Supplementary Materials

Supplementary Materials The CONSORT flow diagram of the study is shown in S-Figure 1. The schematic representation of the study design is provided in S-Figure 2. Positive correlation of plasma hs-CRP levels with glucose AUC during the OGTT and insulin AUC during the 3-hour postprandial period after RE is shown in S-Figure 3. S-Figure 4 illustrates the fiber type, CSA, and myonuclei analyses. The correlation of chymotrypsin-like proteasome activity with hs-CRP and its percent change at 3 hours as well as the enzymatic determination of trypsin-like proteasome activity is shown in S-Figure 5. Information on primary and secondary antibodies used is provided in S-Table 1. S-Table 2 includes effect sizes and confidence intervals for all significant differences observed among groups. S-Table 3 includes data related to participants' daily dietary intake at baseline. The results of fiber composition analysis for ESI and Control groups at baseline are shown in S-Table 4.
