# Supplementary material for: Low-Grade Systemic Inflammation Interferes with Anabolic and Catabolic Characteristics of the Aged Human Skeletal Muscle
Source: Oxid Med Cell Longev. 2021 Dec 7;2021:8376915. doi: 10.1155/2021/8376915 (PMC8670932; doi:10.1155/2021/8376915)
Supplement: Supplementary Materials — The CONSORT flow diagram of the study is shown in S-Figure 1. The schematic representation of the study design is provided in S-Figure 2. Positive correlation of plasma hs-CRP levels with glucose AUC during the OGTT and insulin AUC during the 3-hour postprandial period after RE is shown in S-Figure 3. S-Figure 4 illustrates the fiber type, CSA, and myonuclei analyses. The correlation of chymotrypsin-like proteasome activity with hs-CRP and its percent change at 3 hours as well as the enzymatic determination of trypsin-like proteasome activity is shown in S-Figure 5. Information on primary and secondary antibodies used is provided in S-Table 1. S-Table 2 includes effect sizes and confidence intervals for all significant differences observed among groups. S-Table 3 includes data related to participants' daily dietary intake at baseline. The results of fiber composition analysis for ESI and Control groups at baseline are shown in S-Table 4. [file 8376915.f1.DOC]

|  | **Page** |
| --- | --- |
| **S-Figures** |  |
| S-Figure 1. The CONSORT flow diagram of the study | 2 |
| S-Figure 2. Schematic representation of the study design | 3 |
| S-Figure 3. Positive correlation of plasma hs-CRP levels with glucose AUC during the OGTT and insulin AUC during the 3-hour postprandial period after RE | 3 |
| S-Figure 4. Fiber type, CSA and myonuclei analyses | 4 |
| S-Figure 5. Basal chymotrypsin-like proteasome activity depends on the inflammatory status whereas its post-exercise activation is regulated by its basal activity in a negative feedback loop | 4 |
| **S-Tables** |  |
| S-Table 1. Information on primary and secondary antibodies | 5 |
| S-Table 2. Effect size and confidence intervals for all significant differences observed among groups | 6 |
| S-Table 3. Participants’ daily dietary intake at baseline | 7 |
| S-Table 4. Fiber composition in ESI and Control at baseline | 8 |

**Supplementary Figures**

**
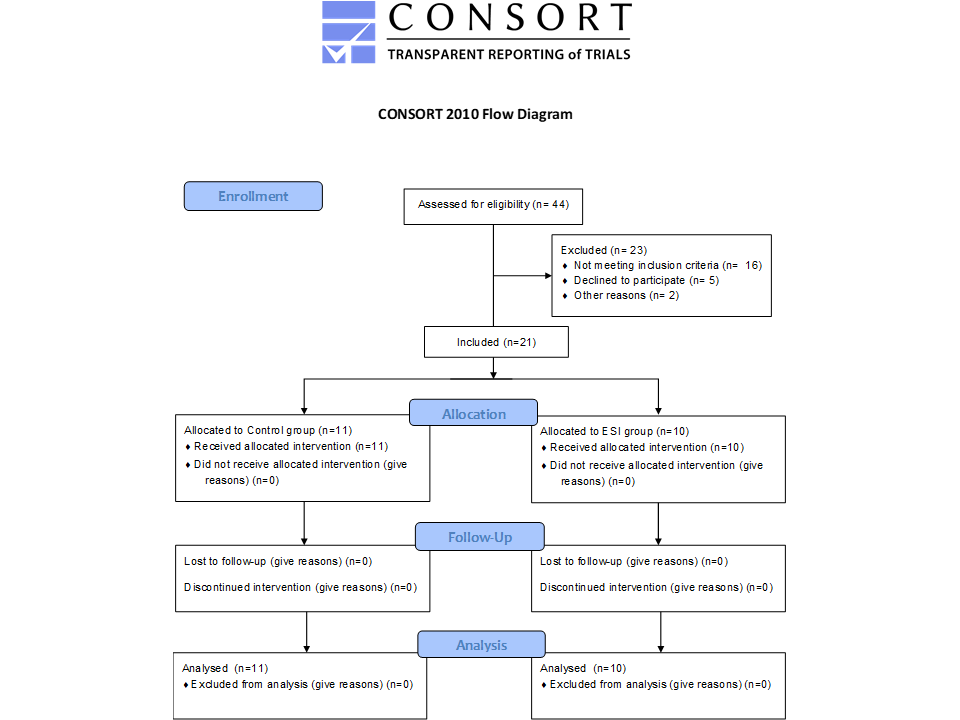
**

**S-Figure 1. The CONSORT flow diagram of the study.**

**
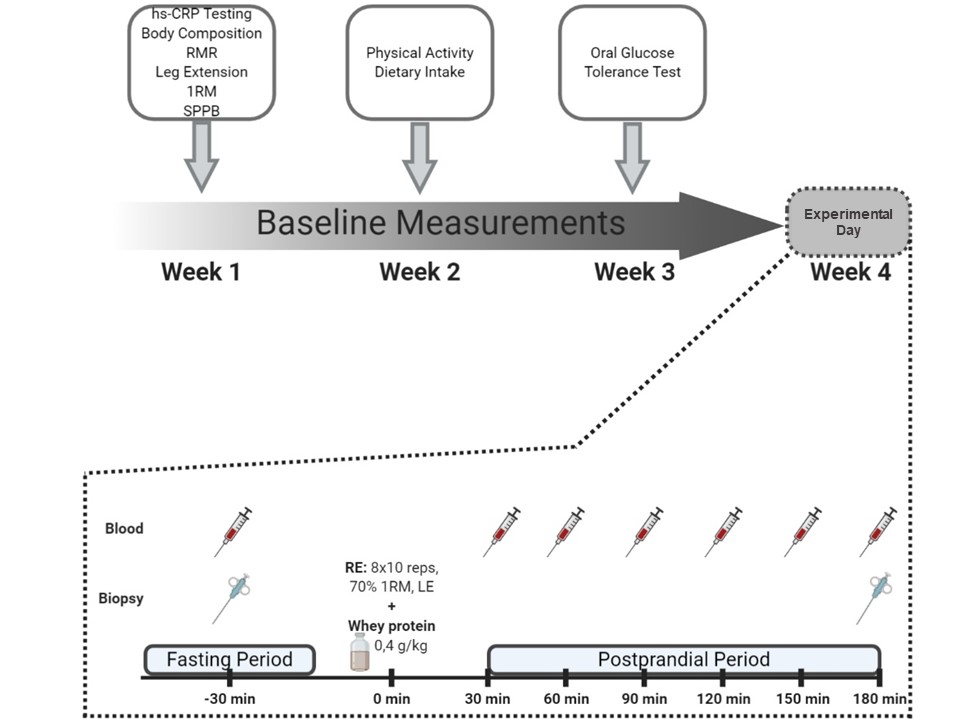
**

**S-Figure 2. Schematic representation of the study design.**

**
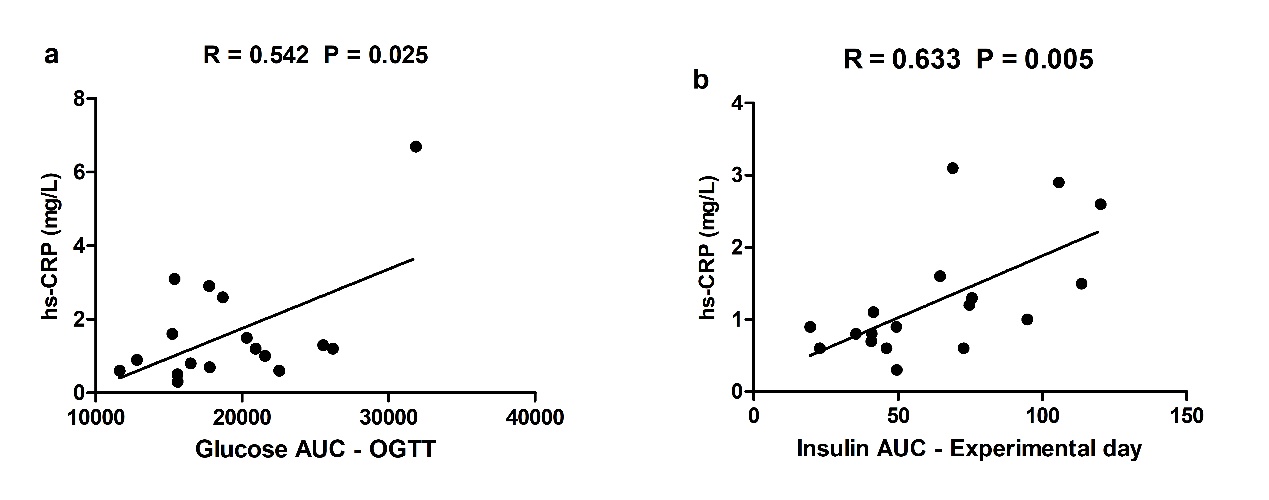
**

**S-Figure 3. Positive correlation of plasma hs-CRP levels with glucose AUC during the OGTT and insulin AUC during the 3-hour postprandial period after RE.** (a) Significant positive correlation between glucose AUC during the OGTT and plasma hs-CRP levels (P = 0.008, R = 0.620) (*N* = 17). (b) Significant positive correlation between insulin AUC during the 3-hour postprandial period after RE (experimental day) and plasma hs-CRP levels (P = 0.005, R = 0.633) (*N* = 18).

**
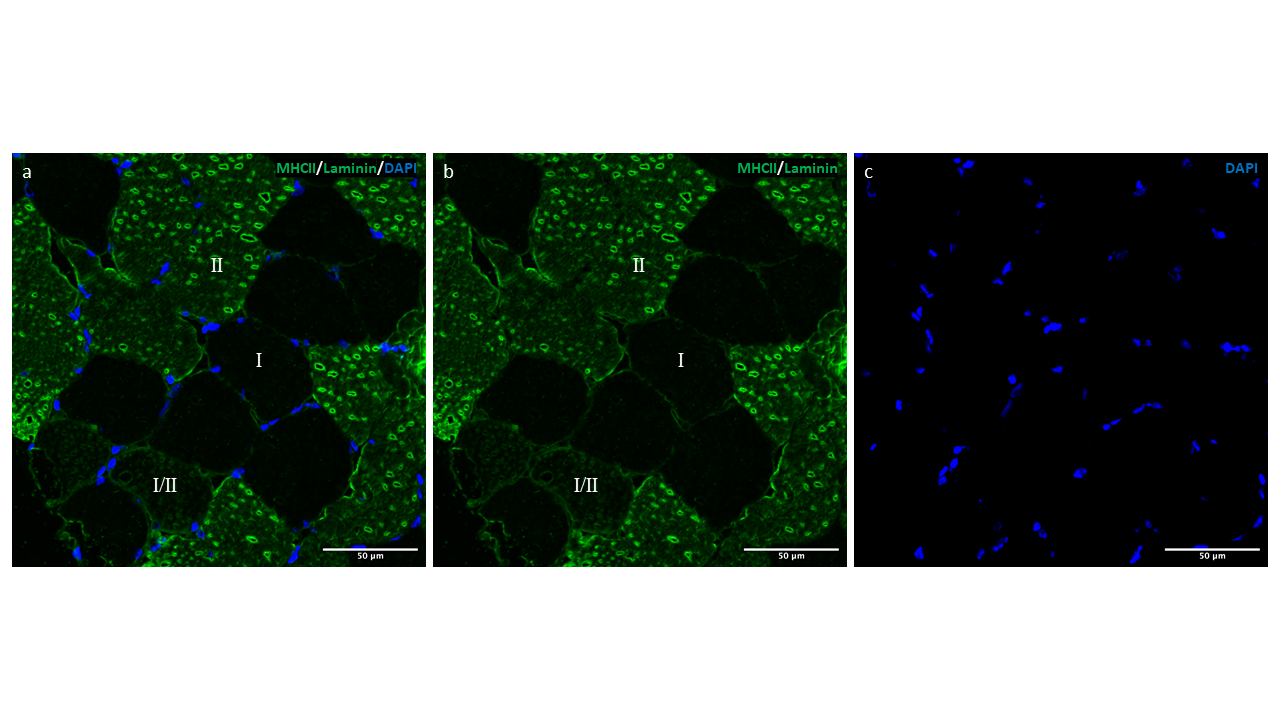
**

**S-Figure 4. Fiber type, CSA and myonuclei analyses.** (a) Representative image of a MHCII/laminin/DAPI staining of a muscle cross section. Single channel images of (b) MHCII/laminin and (c) DAPI. *Numbers* indicate type I, and II muscle fibers. Hybrid fibers (I/II) were excluded from the analyses.


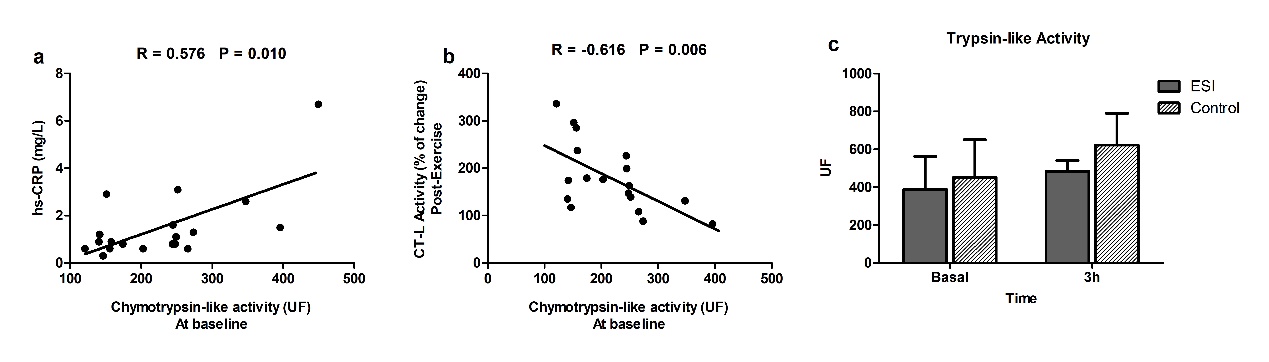


**S-Figure 5. Basal chymotrypsin-like proteasome activity depends on the inflammatory status whereas its post-exercise activation is regulated by its basal activity in a negative feedback loop**. (a) Correlation of chymotrypsin-like activity with hs-CRP levels in the basal state (P = 0.010, R = 0.576) (*N* = 19). (b) Correlation of chymotrypsin-like activity in the basal with its percent change at 3 hours following RE and protein bolus ingestion (P = 0.006, R = - 0.616) (*N* = 18). (**c**) Enzymtic determination of trypsin-like proteasome activity (*N* = 9 in ESI and 10 in Control).

**S-Tables**

| **S-Table 1**. Information on primary and secondary antibodies. | | | | |
| --- | --- | --- | --- | --- |
| **Antibody** | **Type** | **Company** | **Code** | **Dilution** |
| Proteasome 20S β5 | Primary, Polyclonal | Enzo Life Sciences | BML-PW8895 | 1:1000 |
| Proteasome 20S β1i | Primary, Polyclonal | Enzo Life Sciences | BML-PW8345 | 1:1000 |
| Proteasome 20S β2i | Primary, Polyclonal | Enzo Life Sciences | BML-PW8150 | 1:1000 |
| Proteasome 20S β5i | Primary, Polyclonal | Enzo Life Sciences | BML-PW8355 | 1:1000 |
| Phospho IKKα/β | Primary, Monoclonal | Cell Signaling | #2697 | 1:150 |
| Phospho rpS6 | Primary, Monoclonal | Cell Signaling | #4858 | 1:1000 |
| Anti-rabbit IgG, HRP-linked | Secondary, Monoclonal | Cell Signaling | #7074 | 1:2000 |
| Anti-mouse IgG, HRP linked | Secondary, Monoclonal | Cell Signaling | #7076 | 1:2000 |
| LAT1 | Primary, Polyclonal | Cell Signaling | #5347 | 1:1000 |
| c-Myc | Primary, Monoclonal | OriGene Technologies | #TA500003 | 1:1000 |
| SLC38A2 | Primary, Polyclonal | OriGene Technologies | #TA315521 | 1:500 |
| Phospho TIF-1A | Primary, Polyclonal | Sigma-Aldrich | #SAB4504731 | 1:500 |
| Anti-3-Nitrotyrosine | Primary, Monoclonal | Abcam | ab61392 | 1:200 |
| Anti-Nrf2 | Primary, Polyclonal | Abcam | ab137550 | 1:200 |
| Anti-MHCII | Primary, Polyclonal | Abcam | ab91506 | 1:1000 |
| Anti-Laminin | Primary, Polyclonal | Abcam | ab11575 | 1:1000 |
| Anti-PAX7 | Primary, Monoclonal | Developmental Studies Hybridoma Bank (DSHB, USA) | N/A | 1:100 |
| Alexa Fluor 488 goat anti-rabbit | Secondary, Polyclonal | ThermoFisher Scientific | A-11008 | 1:500 |
| Alexa Fluor 488 goat anti-mouse | Secondary, Polyclonal | ThermoFisher Scientific | A-21121 | 1:500 |
| Alexa Fluor 594 goat anti-rabbit | Secondary, Polyclonal | ThermoFisher Scientific | A-11012 | 1:500 |
| Alexa Fluor 594 goat anti-mouse | Secondary, Polyclonal | ThermoFisher Scientific | A-11005 | 1:500 |
| OxyBlot protein oxidation detection kit | N/A | Merck Millipore | S7150 | N/A |

| **S-Table 2**. Effect size and confidence intervals for all significant differences observed among groups. | | | | | |
| --- | --- | --- | --- | --- | --- |
| **Tested Variable** | **ESI**  *Mean ± SD* | **Control**  *Mean ± SD* | ***P* Value** | **Effect Size** | **Confidence Interval** |
| Body Weight | 88.0 ± 4.7 | 81.6 ± 7.4 | 0.029 | 0.98 | [0.07, 1.89] |
| SPPB | 11.5 ± 0.5 | 11.9 ± 0.3 | 0.049 | -0.94 | [-1.85, -0.04] |
| hs-CRP | 2.3 ± 1.7 | 0.6 ± 0.2 | 0.009 | 1.38 | [0.43, 2.34] |
| Glucose AUC *(OGTT)* | 21335.6 ± 5227.5 | 16055.6 ± 3546.3 | 0.035 | 1.08 | [0.05, 2.11] |
| Insulin AUC *(OGTT)* | 10198.0 ± 6101.4 | 5081 ± 1582.9 | 0.048 | 1.01 | [-0.02, 2.03] |
| HOMA-IR *(OGTT)* | 3.0 ± 1.4 | 1.5 ± 0.8 | 0.021 | 1.19 | [0.14, 2.23] |
| ISI_comp_ *(OGTT)* | 3.1 ± 1.1 | 6.9 ± 3.0 | 0.002 | -1.73 | [-2.86, -0.61] |
| Insulin AUC  *(experimental day)* | 84.4 ± 25.8 | 41.9 ± 15.7 | 0.001 | 1.90 | [0.78, 3.01] |
| CT-L Activity  *(Basal)* | 256.9 ± 86.5 | 185.6 ± 51.1 | 0.022 | 0.99 | [0.00, 1.97] |
| Protein Carbonyls  *(Basal)* | 23.2 ± 6.5 | 17.1 ± 8 | 0.026 | 0.79 | [-0.20, 1.78] |
| Phospho IKKα/β (*Basal)* | 14.7 ± 3.2 | 10.7 ± 3.1 | 0.027 | 1.18 | [0.01, 2.36] |
| Phospho rpS6 *(3h)* | 1.3 ± 0.4 | 2.5 ± 1.4 | 0.030 | -1.08 | [-2.09, -0.43] |
| Pax7^+^ cells/Myofiber  *(Basal)* | 0.057 ± 0.009 | 0.072 ± 0.008 | 0.004 | -1.65 | [-2.88, -0.43] |
| Type I fibers CSA *(Basal)* | 5198 ± 860.1 | 4069 ± 656.4 | 0.019 | 1.41 | [0.35, 2.48] |
| Data are presented as mean ± SD. SPPB: short physical performance battery; AUC: area under the curve; OGTT: oral glucose tolerance test; CT-L: chymotrypsin-like activity; CSA: cross sectional area. | | | | | |

| **S-Table 3**. Participants’ daily dietary intake at baseline | | |  |
| --- | --- | --- | --- |
| **Parameter** | **Control** (*N*=11) | **ESI** (*N*=10) | ***t* test** (*p* value) |
| Total energy (kcals) | 1692.8 ± 348.4 | 1743.58 ± 513.1 | 0.083 |
| Protein (g/day) | 67.7 ± 21.8 | 71.5 ± 20.9 | 0.226 |
| Protein (g/kg/day) | 0.8 ± 0.1 | 0.8 ± 0.1 | 0.876 |
| Leucine (g/day) | 5.22 ± 1.8 | 5.47 ± 1.73 | 0.253 |
| BCAA (g/day) | 11.89 ± 4.1 | 12.4 ± 3.8 | 0.270 |
| CHO (g/day) | 160.3 ± 40.6 | 167.1 ± 63.7 | 0.205 |
| Fat (g/day) | 82.9 ± 19.9 | 78.7 ± 25.4 | 0.517 |
| Data are presented as mean ± SD. BCAA: Branched chain amino acids; CHO: Carbohydrates. | | | |

| **S-Table 4**. Fiber composition in ESI and Control at baseline. | | |  |
| --- | --- | --- | --- |
| **Parameter** | **Control** (*N*=9) | **ESI** (*N*=8) | ***t t*est** |
| Total fibers (n) | 161.8 ± 50.7 | 155.4 ± 74.9 | 0.842 |
| Type I fibers (n) | 80.3 ± 36.6 | 91.4 ± 47.1 | 0.570 |
| Type II fibers (n) | 81.4 ± 31.3 | 64.0 ± 28.9 | 0.304 |
| Type I fibers (%) | 51.8 ± 11.8 | 58.7 ± 4.7 | 0.193 |
| Type II fibers (%) | 48.2 ± 11.8 | 41.4 ± 4.7 | 0.193 |
| Total nuclei (n) | 495.6 ± 161.7 | 634.4 ± 275.0 | 0.237 |
| Type I fibers CSA (μm^2^) | 4069 ± 656 | 5198 ± 860 | **0.019** |
| Type II fibers CSA (μm^2^) | 3633 ± 846 | 4359 ± 1040 | 0.168 |
| Myonuclear domain of type I fibers (μm^2^) | 1352 ± 328 | 1202 ± 150 | 0.239 |
| Myonuclear domain of type II fibers (μm^2^) | 1166 ± 216 | 1106 ± 183 | 0.554 |
| Data are presented as mean ± SD. CSA: cross sectional area. ***** Difference between groups, P < 0.05. | | |  |
